# Evaluation of Reported Fertility Preservation Counseling Before Chemotherapy Using the Quality Oncology Practice Initiative Survey

**DOI:** 10.1001/jamanetworkopen.2020.10806

**Published:** 2020-07-17

**Authors:** Premal Patel, Taylor P. Kohn, Jordan Cohen, Benjamin Shiff, Jaden Kohn, Ranjith Ramasamy

**Affiliations:** 1 Section of Urology, University of Manitoba, Winnipeg, Manitoba, Canada; 2 The James Buchanan Brady Urological Institute and Department of Urology, Johns Hopkins University School of Medicine, Baltimore, Maryland; 3 Department of Urology, University of Miami Miller School of Medicine, Miami, Florida; 4 Department of Gynecology and Obstetrics, Johns Hopkins University School of Medicine, Baltimore, Maryland

## Abstract

**Importance:**

The opportunity to discuss fertility preservation is essential for patients of reproductive age with newly diagnosed cancer before the initiation of treatment.

**Objective:**

To identify factors associated with fertility preservation counseling among patients of reproductive age before initiating chemotherapy.

**Design, Setting, and Participants:**

This cross-sectional study used data obtained from the American Society of Clinical Oncology (ASCO) Quality Oncology Practice Initiative, an oncologist-led quality assessment program that surveys approximately 400 oncology practices biannually, from January, 2015, to June, 2019.

**Main Outcomes and Measures:**

The primary outcome was whether reproductive risks were discussed before initiation of chemotherapy. Multivariate logistic regression was performed to identify factors associated with fertility preservation counseling, controlling for age, sex, race/ethnicity, cancer type, year of study, region, clinic type (academic vs private), annual clinic volume, and rates of insurance coverage.

**Results:**

Among the 6976 patients of reproductive age (3571 men [51%]; mean (SD) age, 42.5 [7.1] years), with reproductive age ranging from 18 to 40 years for 3405 women and from 18 to 50 years for 3571 men, clinics reported that 3036 of 6976 patients (44%) received counseling regarding the risk of infertility associated with chemotherapy. Women were more likely to be informed (1912 of 3405 [56%]) compared with men (1126 of 3571 [32%]) (*P* < .001). Factors associated with reduced likelihood of fertility risk discussion included male sex (odds ratio [OR], 0.73; 95% CI, 0.60-0.90), increasing age (OR, 0.93; 95% CI, 0.92-0.94), private practice setting (OR, 0.70; 95% CI, 0.53-0.93), and lack of multidisciplinary team planning (OR, 0.54; 95% CI, 0.41-0.70). Factors associated with increased likelihood of fertility risk discussion included having breast cancer (OR, 1.39; 95% CI, 1.12-1.73) and lymphatic or hematopoietic cancers (OR, 1.79; 95% CI, 1.33-2.40), participating in each subsequent study year (OR, 1.16; 95% CI, 1.08-1.24), receiving care in an academic clinic (OR, 1.45; 95% CI, 1.05-2.01), and being a practice offering clinical trial enrollment (OR, 1.60; 95% CI, 1.13-2.29). States with legislatively mandated coverage of fertility preservation had significantly higher rates of fertility risk discussion compared with states without legislation (48.6% vs 39.6%, *P* < .001).

**Conclusions and Relevance:**

The findings suggest that clinicians are more likely to counsel younger patients and female patients about reproductive risks before initiation of chemotherapy. State laws mandating fertility preservation coverage may be associated with improved frequency of fertility counseling before chemotherapy. Further awareness and implementation of ASCO guidelines appear to be needed to improve rates of fertility risk discussion and referrals to fertility specialists before chemotherapy.

## Key Points

**Question:**

What factors were associated with discussions about fertility preservation before initiating chemotherapy and referrals to reproductive specialists among patients with recently diagnosed cancer?

**Findings:**

In this cross-sectional study of 6976 patients of reproductive age, 43.5% had a discussion with their clinician about the risk of infertility associated with chemotherapy; women were more likely to be counseled. Discussions of fertility preservation occurred at higher rates in academic centers compared with private practice settings, and mandated state laws for fertility preservation were associated with improved frequency of discussing fertility risks.

**Meaning:**

The findings suggest that the American Society of Clinical Oncology quality measures should be better disseminated for patients of reproductive age regarding discussion of infertility risk and fertility preservation and appropriate referral to fertility specialists before the onset of chemotherapy.

## Teaser

This cross-sectional study evaluates factors associated with fertility preservation counseling before initiating chemotherapy among patients of reproductive age with recently diagnosed cancer.

## Introduction

As cancer survival increases, quality of life is becoming increasingly important to patients, with fertility preservation of significant importance for both men and women. Whereas chemotherapy and radiotherapy are effective in eliminating cancer cells, adverse effects on the reproductive system persist. In males, this toxic environment can directly damage the germinal epithelium, causing azoospermia or oligozoospermia, or can compromise the neural pathway responsible for erection and ejaculation.^1^ In females, cancer therapies can deplete both the number and integrity of oocytes.^2,3^

The American Society for Reproductive Medicine and the American Society of Clinical Oncology (ASCO) recommend that health care clinicians caring for adult and pediatric patients with cancer discuss fertility preservation before the initiation of anticancer therapy.^4,5,6,7^ Although the initial guidelines were published in 2006, ASCO has since released 3 iterations; the most recent guidelines were published in 2018.^7^ Current guidelines recommend that clinicians address the possibility of infertility as early as possible before treatment starts and refer to an appropriate specialist for patients who express an interest in fertility preservation. Whether a patient is a candidate for or chooses to pursue fertility preservation, having this discussion may be associated with reduced distress and improved quality of life. A study^6^ evaluating the ASCO Quality Oncology Practice Initiative (QOPI), a nationwide database reporting 150 quality measures, found that only 6% of US patients had a fertility preservation discussion before undergoing chemotherapy between 2006 and 2010. In recent years, there has been increased effort to improve the rates of fertility counseling before chemotherapy, including increased education for oncologists regarding the risks of infertility associated with chemotherapy, additional updates of ASCO guidelines, and an increased number of states passing legislative initiatives requiring insurance carriers to cover fertility preservation for those requiring chemotherapy.^7,8^ Given these efforts to improve fertility counseling, we used recent data from the ASCO QOPI to assess current rates of fertility counseling in reproductive-aged patients and evaluated factors associated with discussion of fertility risks and fertility preservation before initiation of chemotherapy.

## Methods

This cross-sectional study used data from the ASCO QOPI, which surveys approximately 400 clinical practices yearly, covering 150 evidence-based quality measures. Reporting by clinics is voluntary and not all measures are reported annually. An ethics waiver was obtained from the University of Miami institutional research board, Miami, Florida, because anonymized data were procured from the ASCO QOPI data set. Informed consent to be included in the data set was obtained by participating clinical practices. This study followed the Strengthening the Reporting of Observational Studies in Epidemiology (STROBE) reporting guideline for a cross-sectional analysis.^9^

The ASCO QOPI engages in 2 data collection rounds per year; however, practices are not required to participate in both rounds. Approximately 200 to 250 clinics participate in 1 round per year, with some clinics participating in both rounds in a year. Specific fertility quality measures include whether the following occurred before initiation of chemotherapy: (1) infertility risks associated with chemotherapy were discussed, (2) fertility preservation options were discussed, or (3) referral was made to a reproductive specialist. Patients of reproductive age were defined as women between the ages of 18 and 40 years and men between the ages of 18 and 50 years. Our analysis included patients who were defined as being of reproductive age, received a diagnosis of a cancer that required chemotherapy, and eventually underwent chemotherapy. Data from clinical practices within the US from January, 2015, to June, 2019, were included in our analysis. Patients with a history of infertility or purposeful sterilization were excluded from the analysis.

The ASCO QOPI follows strict protocols to ensure quality assurance of their data that are received from certified institutions. Two rounds of data acquisition were conducted at the respective center with at least 2 reviewers to ensure data were acquired correctly. A clinician knowledgeable in the QOPI measures was also assigned from each specialty to assist in the data acquisition process. Surveyors looked for documentation that the patient had received information about adverse effects, including infertility risks.^10^ To assess whether fertility counseling had occurred, surveyors assessed documentation on whether patients had received information about adverse effects associated with chemotherapy, including infertility. Examples of documentation detailing a risk of infertility included documentation within the electronic medical record, consent forms, treatment plans, and/or drug information sheets on given drugs highlighting serious reactions that require contacting the practice and discussing the risk of treatment, including prevention and management. Discussion of infertility risk or referral to a fertility specialist was determined if such discussion was documented or a referral was made.^11^

Patient-level data available included sex, age, race/ethnicity, type of cancer, year of inclusion, whether the patient underwent discussion regarding infertility risk associated with chemotherapy, and whether the patient was counseled regarding fertility preservation options or referral to a reproductive specialist. In addition to patient-level variables, data were available for each clinical practice. Practice-level data included (1) the number of clinical sites per practice; (2) the region of the practice; (3) the number of new patients seen per year; (4) academic status of the practice; (5) the number of oncology attending physicians, nurse practitioners, and physician assistants per practice; (6) the percentage of patients with commercial insurance, Medicare, Medicaid, or no insurance; (7) the percentage of patients for whom English was not their primary language; (8) whether the practice offered multiple oncologic specialties; (9) whether the practice participated in quality improvement planning; (10) whether the practice participated in multidisciplinary team planning; and (11) whether the practice offered clinical trial enrollment for cancer therapies.

The primary objective of this study was to assess whether any patient or practice-level characteristic was associated with increased prevalence of fertility counseling. Given the complex association between these variables, a multivariate logistic regression was performed controlling for the above variables.

Two secondary analyses were subsequently performed. The first analysis assessed the association of counseling with subsequent referral to a fertility specialist. The second analysis assessed the association of state legislative changes requiring insurance coverage of fertility preservation with changes in counseling and referral to fertility specialists. Legislative fertility preservation policies for each state were found on Resolve.org.^12^

### Statistical Analysis

The Kolmogorov-Smirnov test was used to assess normality of numerical variables. Univariate comparative statistics were performed using the Mann-Whitney test and Fisher exact test when appropriate. Multivariate logistic regression was performed using R, version 3.4.1 (R Foundation for Statistical Computing). Statistical significance was 2-sided and set at *P* < .05.

## Results

A total of 136 746 charts were reviewed, with 6976 patients (3571 men [51%]; mean [SD] age, 42.5 [7.1] years) identified as being of reproductive age (Table 1 and Table 2). Overall, 3036 of 6976 patients (44%) were counseled about the risks of infertility associated with chemotherapy. Women were more likely to be counseled about the risk of infertility (1912 of 3405 [56%]) compared with men (1126 of 3571 [32%]; *P* < .001) (Figure, A and Table 3). The frequency of discussion about the risks of infertility decreased as patient age increased (OR, 0.91; 95% CI, 0.90-0.92; *P* < .001) (Figure, B). There was no difference with respect to patient race/ethnicity (black: OR, 1.03 [95% CI, 0.84-1.27]; Asian: OR, 1.14 [95% CI, 0.78-1.69]; Hispanic: OR, 1.04 [95% CI, 0.86-1.26]; other or not reported: OR, 0.99 [95% CI, 0.83-1.17]) (Figure, C). Patients from the Northeast were more likely to have infertility risks discussed before initiating chemotherapy (OR, 1.89; 95% CI, 1.43-2.51; *P* < .001) compared with those from other regions (South: OR 1.26 [95% CI, 0.96-1.64]; West: OR, 1.09 [95% CI, 0.82-1.45]; Midwest: OR, 1.23 [95% CI, 0.95-1.60]; *P* < .001) (Figure, D). During the study period, the rate of infertility risk discussion increased (OR, 1.02; 95% CI, 1.01-1.02; *P* < .001) (Figure, E).

**Table 1.** Baseline Patient Characteristics

Characteristic	Patients, No. (%)		
	Total (N = 6976)	Female (n = 3405)	Male (n = 3571)
Age, y			
18-25	288 (4)	176 (5)	112 (3)
26-30	652 (9)	460 (14)	192 (5)
31-35	1392 (20)	1072 (31)	320 (9)
36-40	2191 (31)	1697 (50)	494 (14)
41-45	817 (12)	0	817 (23)
46-50	1636 (24)	0	1636 (46)
Race/ethnicity			
White	4147 (59)	1903 (56)	2244 (63)
Black	811 (12)	409 (12)	402 (11)
Asian	191 (3)	109 (3)	82 (2)
Hispanic	820 (12)	424 (12)	396 (11)
Other or not reported	1007 (14)	560 (17)	447 (13)
Region			
South	1912 (27)	928 (27)	984 (28)
West	1023 (15)	515 (15)	508 (14)
Northeast	1552 (22)	770 (23)	782 (22)
Midwest	2489 (36)	1192 (35)	1297 (36)
Period			
Fall 2015	792 (11)	382 (11)	410 (11)
Spring 2016	1083 (16)	500 (15)	583 (16)
Fall 2016	939 (13)	448 (13)	491 (14)
Spring 2017	968 (14)	495 (15)	473 (13)
Fall 2017	1052 (15)	489 (14)	563 (16)
Spring 2018	1052 (15)	519 (15)	533 (15)
Fall 2018	1028 (15)	542 (16)	486 (14)
Spring 2019	62 (1)	30 (1)	32 (1)
Type of cancer			
Bone or skin	61 (1)	15 (0.44)	46 (1)
Breast	2229 (32)	2219 (65)	10 (0.28)
Ear, nose, and throat	50 (1)	5 (0.15)	45 (1)
Gastrointestinal	2509 (36)	475 (14)	2034 (57)
Genitourinary or reproductive	238 (3)	109 (3)	129 (4)
Lymphatic or hematopoietic	434 (6)	113 (3)	321 (9)
Neurologic	19 (0.27)	5 (0.15)	14 (0.39)
Thoracic	568 (8)	65 (2)	503 (14)
Other or not reported	868 (12)	399 (12)	469 (13)

**Table 2.** Demographic Practice Data

Variable	Total (N = 448)	Academic (n = 74)	Private (n = 361)
Clinical sites per practice, median (IQR)	1 (1-3)	1 (1-3)	1 (1-3)
New patients seen per practice per year, median (IQR)	1100 (503-2200)	2598 (899-5400)	987 (500-1746)
Clinicians per practice, median (IQR)			
Medical oncologists	2 (0-7)	11 (1-25)	1 (0-5)
Hematologist oncologists	3 (0-7)	7 (3-13)	2 (0-6)
Radiation oncologists	1 (0-3)	5 (0-11)	1 (0-2)
Physician assistants	0 (0-2)	1 (0-6)	0 (0-1)
Nurse practitioners	2 (1-5)	6 (1-18)	2 (1-4)
Insurance coverage, mean (IQR)			
Private insurance	40 (30-50)	40 (27-50)	40 (30-50)
Medicare	47 (36-55)	39 (29-47)	50 (40-56)
Medicaid	8 (5-12)	13 (5-22)	8 (5-10)
Uninsured	2 (1-5)	3 (1-10)	2 (1-5)
Non-English speakers, mean (IQR)	1 (1-2)	1 (1-2)	1 (1-1)
Multispecialty site, No. (%)			
Yes	255 (57)	51 (69)	195 (54)
No	193 (43)	23 (31)	166 (46)
Quality improvement staff, No. (%)^a^			
Yes	304 (68)	60 (81)	237 (66)
No	139 (31)	14 (19)	124 (34)
Multidisciplinary team planning, No. (%)^b^			
Yes	418 (93)	73 (99)	337 (93)
No	26 (6)	1 (1)	24 (7)
Offers clinical trials enrollment, No. (%)			
Yes	392 (88)	71 (96)	310 (86)
No	56 (12)	3 (4)	51 (14)
Patients enrolled per year, median (IQR)	48 (20-145)	294 (71-542)	40 (15-80)

Abbreviation: IQR, interquartile range.

^a^ Data were missing for 5 locations.

^b^ Data were missing for 4 locations.

**Figure.**
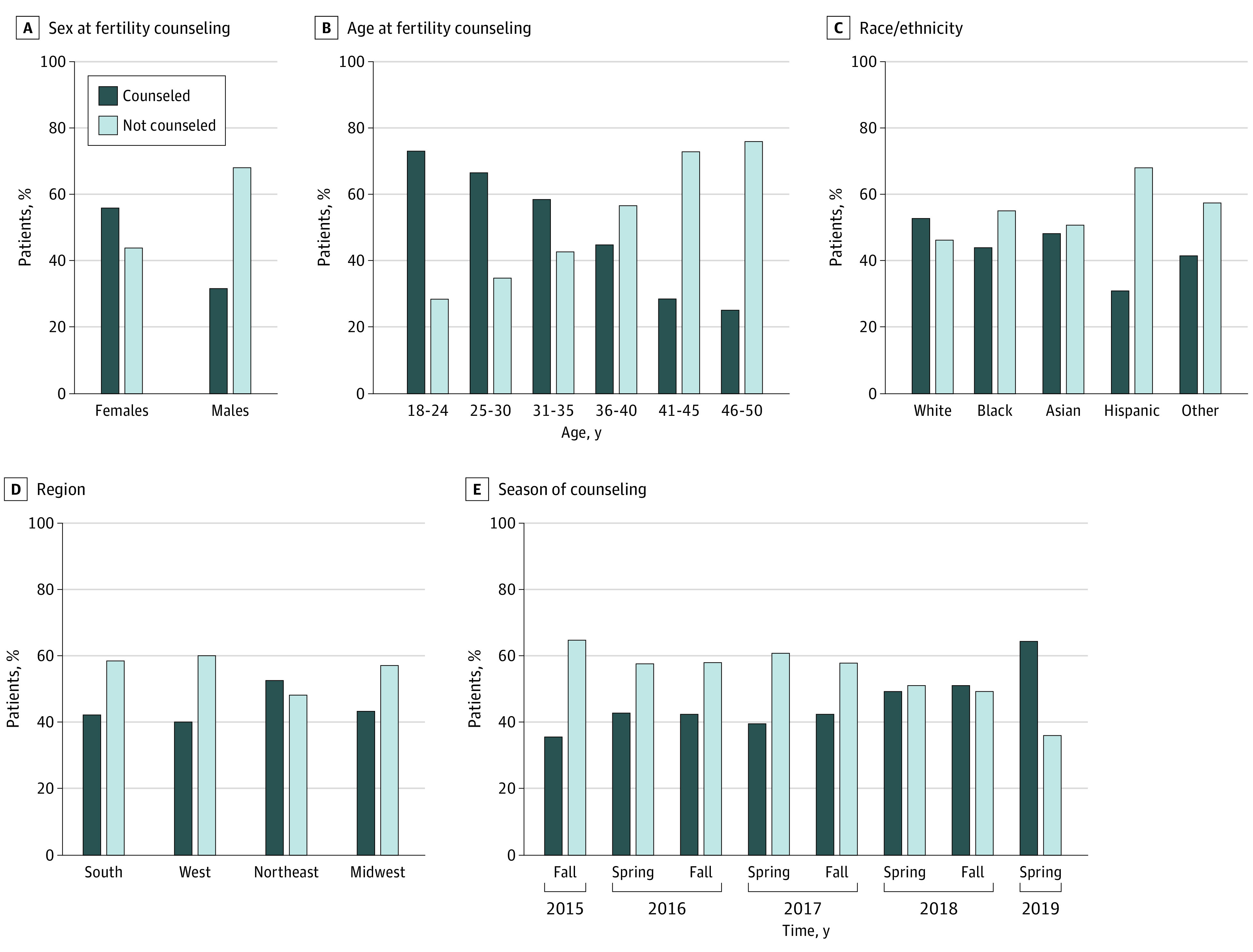
Association of Sex, Age, Race/Ethnicity, Region, and Time With Fertility Counseling Before Initiation of Chemotherapy

**Table 3.** Multivariate Regression of Patient-Level Factors

Variable	Total patients, No.	Patients counseled, No. (%)	Odds Ratio (95% CI)
Sex			
Female	3405	1912 (56)	1 [Reference]
Male	3571	1126 (32)	0.73 (0.60-0.90)
Age, y			
18-25	288	208 (72)	1 [Reference]
26-30	652	428 (66)	0.70 (0.49-0.99)
31-35	1392	803 (58)	0.53 (0.38-0.72)
36-40	2191	967 (44)	0.29 (0.21-0.40)
41-45	817	228 (28)	0.23 (0.16-0.33)
46-50	1636	404 (25)	0.21 (0.14-0.29)
Race/ethnicity			
Caucasian	4147	1822 (44)	1 [Reference]
Black	811	363 (45)	1.03 (0.84-1.27)
Asian	191	93 (49)	1.14 (0.78-1.69)
Hispanic	820	434 (53)	1.04 (0.86-1.26)
Other or not reported	1007	326 (32)	0.99 (0.83-1.17)
Region			
South	1912	798 (42)	1.26 (0.96-1.64)
West	1023	406 (40)	1.09 (0.82-1.45)
Northeast	1552	771 (50)	1.89 (1.43-2.51)
Midwest	2489	1063 (43)	1.23 (0.95-1.60)
Type of cancer			
Bone or skin	61	22 (36)	0.55 (0.28-1.03)
Breast	2229	1325 (59)	1.39 (1.12-1.73)
Ear, nose, and throat	50	16 (32)	0.74 (0.35-1.48)
Gastrointestinal	2509	796 (32)	0.77 (0.62-0.94)
Genitourinary or reproductive	238	112 (47)	0.92 (0.63-1.33)
Lymphatic or hematopoietic	434	251 (58)	1.79 (1.33-2.40)
Neurologic	19	6 (32)	0.80 (0.23-2.48)
Thoracic	568	170 (30)	0.85 (0.64-1.12)
Other or not reported	868	340 (39)	0.60 (0.35-1.01)

In multivariate logistic regression, the following factors were associated with reduced likelihood that the fertility risks associated with chemotherapy were discussed (Table 3 and Table 4): male sex (odds ratio [OR], 0.73; 95% CI, 0.60-0.90), older age (overall: OR, 0.93; 95% CI, 0.92-0.94; 36-40 years: OR, 0.29; 95% CI, 0.21-0.40; 41-45 years: OR, 0.23; 95% CI, 0.16-0.33; 46-50 years: OR, 0.21; 95% CI, 0.14-0.29), gastrointestinal malignancy (OR, 0.77; 95% CI, 0.62-0.94), private practice setting (OR, 0.70; 95% CI, 0.53-0.93), increasing prevalence of patients with Medicare (OR, 0.99; 95% CI, 0.99-1.00), practice offering multiple oncologic specialties (OR, 0.70, 95% CI, 0.61-0.81), and lack of multidisciplinary team planning (OR, 0.54, 95% CI, 0.41-0.70).

**Table 4.** Multivariate Logistic Regression of Practice-Level Factors^a^

Variable	Odds Ratio (95% CI)
Clinical sites per practice	1.02 (1.01-1.03)
New patients seen per practice per year	1.00 (1.00-1.00)
Year of inclusion	1.16 (1.08-1.24)
Type of practice	
Academic	0.86 (0.64-1.15)
Private	0.70 (0.53-0.93)
Providers per practice	
Medical oncologists	1.00 (1.00-1.01)
Hematologist oncologist	0.99 (0.99-1.00)
Radiation oncologists	0.99 (0.97-1.01)
Physician assistants	1.00 (1.00-1.00)
Nurse practitioners	1.00 (1.00-1.00)
Insurance coverage	
Private insurance	1.00 (1.00-1.01)
Medicare	0.99 (0.99-1.00)
Medicaid	1.00 (0.99-1.00)
Uninsured	1.00 (0.99-1.01)
Non-English speakers	1.07 (0.93-1.23)
Multispecialty site	
Yes	0.70 (0.61-0.81)
No	1 [Reference]
Quality improvement staff	
Yes	1.24 (0.98-1.63)
No	1 [Reference]
Multidisciplinary team planning	
Yes	1 [Reference]
No	0.54 (0.41-0.70)
Offer clinical trials enrollment	
Yes	1.60 (1.13-2.29)
No	1 [Reference]

^a^ In addition, controlling for number of clinical sites per practice, new patients seen per year, year of inclusion, academic vs private practice, number of clinicians per practice, percent of insurance, percent non-English speakers, whether the practice offered multispecialties, quality improvement planning, multidisciplinary team planning, and whether the practice offers clinical trial enrollment.

The following factors were associated with increased likelihood that the fertility risks associated with chemotherapy were discussed (Table 3 and Table 4): breast cancer (OR, 1.39; 95% CI, 1.12-1.73), lymphatic or hematopoietic cancers (OR, 1.79; 95% CI, 1.33-2.40), practice located in the Northeast (OR, 1.89; 95% CI, 1.43-2.51), each subsequent study year (OR, 1.16; 95% CI, 1.08-1.24), receiving care in an academic clinic (OR, 1.45; 95% CI, 1.05-2.01), and practice offering clinical trial enrollment (OR, 1.60; 95% CI, 1.13-2.29).

Of 940 patients younger than 30 years (the group with the highest future fertility potential), 636 (67.7%) were counseled about the risk of infertility associated with chemotherapy and 523 (55.6%) were subsequently told about fertility preservation options or referred to a reproductive specialist. Of the 304 patients in this group who were not counseled about the risk of impaired fertility associated with chemotherapy, only 32 (10.5%) discussed fertility preservation or were referred to a reproductive specialist.

In 2018, 4 states began legislatively mandated coverage of fertility preservation: Rhode Island, Connecticut, Delaware, and Maryland. In 2019, Illinois joined in legislatively mandated coverage of fertility preservation. In states with legislatively mandated coverage of fertility preservation, patients who were counseled about the risk of infertility were more often counseled regarding fertility preservation or were referred to a fertility specialist after passage of mandated coverage (before legislation: 68 of 95 patients [71.6%]; after legislation: 21 of 26 patients [80.8%]). For states without legislatively mandated coverage of fertility preservation, there was a slight improvement in the rate of discussion of fertility preservation or referral to a fertility preservation clinic after passage of mandated coverage in other states (before legislation in other states: 1189 of 1872 patients [63.5%]; after legislation in other states: 708 of 1045 patients [67.8%]).

## Discussion

Chemotherapy may be associated with patients’ future fertility; thus, counseling patients about these risks before initiation of chemotherapy is necessary. The current study was the largest evaluation to date on whether infertility risk was discussed and preservation options were presented to reproductive age adults antecedent to cancer therapy. Of the 6976 patients in this cross-sectional study using a national quality database, we found that 43.5% of reproductive age patients had a discussion regarding the risk of infertility associated with cancer treatment, despite ASCO recommendations.^7^ We hypothesized that key factors associated with variability in fertility rate discussions were sex, age, type of center in which the patient received care (private, academic, or multispecialty), and percentage of insurance coverage within a clinic. All of these factors had significant associations with variability in these discussions.

Our findings were consistent with a recent survey of 167 female adolescents and young adults from Japan that demonstrated that only 48% of patients with nonbreast and nonhematologic malignancies had received information about the association of cancer therapy with reproductive organ health.^13^ This finding suggests that there exists a need both in the US and elsewhere to identify strategies to increase fertility education and indicated referrals to fertility specialists for adult patients with newly diagnosed cancers.

As expected, counseling and referral were more common in the academic rather than the private clinic setting. Academic institutions might facilitate a more rapid collaboration between oncologists and reproductive specialists. Stemming from this notion of more rapid collaboration, several other variables proved to accompany higher rates of fertility counseling. Specifically, associated factors included whether patients were cared for in multispecialty clinics, were cared for through multidisciplinary team planning, or were seen in a clinic that also offered clinical trials. Surprisingly, multispecialty clinics had reduced rates of fertility risk counseling compared with single specialty clinics. One theory for this decreased rate of counseling in multispecialty clinics could be an unclear delineation of who is responsible for discussing the risk of infertility associated with chemotherapy. A recent qualitative study by Covelli et al^14^ included 22 Canadian physicians who were interviewed regarding adherence to ASCO guidelines about fertility preservation conversations with patients. Multiple physicians in this study^14^ expressed that they did not believe that they were qualified or knew enough about fertility preservation to have conversations about this issue. This study^14^ found that often physicians attributed the responsibility of fertility counseling to other clinicians, focused on the part of the body being treated, and did not see fertility preservation as their responsibility. In these multispecialty clinics, physicians possibly deferred these conversations, assuming that colleagues would address risks, potentially allowing such conversations to be missed.

Our analysis also found that clinics in which clinical trials were offered had higher rates of discussions regarding fertility risks associated with chemotherapy, even when controlling for private vs academic status. These clinics offering clinical trials may be more familiar with more rigorous oversight, informed consent, and internal institutional guidelines, thus facilitating increased rates of counseling.

Although oncologists likely understand the association of chemotherapy and radiotherapy with fertility, many may be unaware of contemporary fertility preservation options.^7,14^ In an interview with oncologists, Covelli et al^14^ found that clinicians felt an absence of confidence in their ability to engage in fertility preservation discussions, often feeling uncomfortable when patients had questions.^14^ Ongoing educational alignment between oncologists and fertility specialists may be of value as a strategy to help practices meet ASCO recommendations. Furthermore, additional research should investigate whether unique barriers exist in private practice oncology settings, followed by undertaking quality improvement initiatives.

We demonstrated that infertility risks were discussed infrequently with patients of reproductive age before chemotherapy despite ASCO guidelines. Protecting options for future parenthood at the time of diagnosis with cancer and appropriate referral to a reproductive specialist should be part of the routine practice of oncologists. Quality improvement initiatives to improve rates of fertility risk discussion and referrals for fertility preservation should be undertaken by clinicians in facilities who are currently deficient on these metrics, and the routine use of ASCO QOPI quality data could track improvements made after implementation of these initiatives. Quality initiatives may include prompts on electronic medical records for patients meeting criteria for fertility discussion and/or referral.

Moreover, we demonstrated that clinicians were more likely to counsel younger patients and female patients. Thus, efforts should be undertaken to reduce these biases to ensure equitable care of patients. States with mandated coverage for oncofertility preservation had improved rates of fertility discussion and referral. Therefore, we encourage advocacy efforts to ensure passage of similar laws in other states to improve patient access to reproductive health.

### Strengths and Limitations

This study has strengths. This study represents, to our knowledge, the largest sample of reproductive-aged patients from a national database. The ASCO QOPI database provides numerous patient and clinic-level variables, allowing multiple factors to be assessed in our multivariable analysis. Furthermore, the ASCO QOPI uses strict protocols to ensure quality of reported data. Clinical sites undergo an extensive application and trial period with multiple rounds of reviewers assessing submitted data.^10,11^ In addition, data included in this study were recent because study years ranged from 2015 to early 2019. Thus, these data are clinically relevant and representative of the present clinical situation.

This study has limitations. Data pertaining to reproductive history of patients (eg, number of children) were unknown, and this may be an important factor associated with whether infertility risks or fertility preservation options were discussed. Although the ASCO QOPI uses multiple layers of review to ensure quality of the data, infertility risk and preservation counseling are self-reported from oncology practices and are subject to error. Discussion of infertility risks may have occurred without medical record documentation, or documentation may have occurred without discussion. Our data only represent whether counseling occurred, but do not fully capture the scope or extent of these conversations. Furthermore, data about patients younger than 18 years are not collected in the ASCO QOPI database, limiting analysis of a group of patients for whom fertility counseling is critical. Data are also not collected for those undergoing radiotherapy or surgery instead of chemotherapy, a subset of oncologic patients for whom fertility discussion is also pertinent. In addition, because chemotherapeutic regimens and fertility referrals were chosen by the physician, it was unclear whether the infertility risks associated with a selected chemotherapeutic regimen were associated with whether fertility referral was provided and with clinic accessibility to a fertility center.

## Conclusions

The findings suggest that clinicians are more likely to counsel younger patients and female patients about reproductive risks before initiation of chemotherapy. State laws mandating fertility preservation coverage may be associated with improved frequency of fertility counseling before chemotherapy. Further awareness and implementation of ASCO guidelines appear to be needed to improve rates of fertility risk discussion and referrals to infertility specialists before chemotherapy.
